# The Phytochemical Potential of *Viola* Species, *Melanium* Subgenus, Subsection *Bracteolatae*

**DOI:** 10.3390/ijms262311614

**Published:** 2025-11-30

**Authors:** Elida Rosenhech, Andrei Lobiuc, Irina Boz, Maria-Magdalena Zamfirache

**Affiliations:** 1 Faculty of Biology, “Alexandru Ioan Cuza” University of Iași, 700506 Iași, Romania; elidarosenhech@gmail.com (E.R.);; 2 Faculty of Medicine and Biological Sciences, “Stefan cel Mare” University, 720229 Suceava, Romania; irina.boz@usm.ro

**Keywords:** phenols, flavonoids, antioxidant properties, *Viola*, *Melanium*

## Abstract

This study provides a comparative phytochemical evaluation of five *Viola* L. species belonging to the *Melanium* subgenus, subsection *Bracteolatae: V. declinata*, *V. dacica*, *V. tricolor*, *V. arvensis*, and *V. kitaibeliana*. Plant material was collected from natural populations in northeastern Romania, encompassing both alpine and agricultural habitats. We analyzed assimilatory pigments, anthocyanins, total phenolics, flavonoids, and antioxidant activity from aqueous and 50% ethanolic extracts (four plant-to-solvent ratios). Results showed that *V. dacica* and *V. declinata*, two Carpathian endemic taxa, had the highest contents of anthocyanins and phenolic compounds, which strongly correlated with their antioxidant activity. The quantitative analyses of total phenols for *V. dacica* were up to 94.81 ± 1.37 mg gallic acid equivalents/gram (GAE/g) (1% hydroalcoholic extract), and for anthocyanins were up to 113 ± 0.128 mg/g. Meanwhile, for *V. declinate*, total phenols were up to 95.53 ± 0.33 mg GAE/g (1% hydroalcoholic extract), and for anthocyanins, were up 0.909 ± 0.054 mg/g. *V. kitaibeliana* extracts, although obtained from lowland populations, were distinguished by elevated flavonoid concentrations, up to 56.61 ± 1.19 mg quercetin equivalents/gram (QE/g) dry biomass (0.5% hydroalcoholic extract). In contrast, *V. arvensis* and *V. tricolor*, cosmopolitan and widely used in traditional medicine, exhibited lower levels of bioactive compounds under the same extraction conditions. These findings highlight the pharmaceutical and ecological potential of less-studied *Viola* species and provide the first quantitative comparative data for *V. dacica*, *V. declinata*, and *V. kitaibeliana*. The results support the valorization of these taxa as valuable sources of bioactive secondary metabolites with antioxidant properties.

## 1. Introduction

The genus *Viola* L. comprises approximately 600 species distributed globally, ranging from temperate zones to the tropics. Most are perennial herbaceous plants, with rare occurrences of annual herbs and shrubs [1]. Of these, 28 species grow spontaneously in Romania [2,3]. The genus is divided into roughly 16 subgenera worldwide [4]; however, only five—*Delphiniopsis* W. Becker, *Melanium* Ging., *Plagiostigma* Godr., *Viola* s. str., and *Xylinosium* W. Becker—contain species native to Eurasia [4,5].

Historically, *Viola* species have attracted scientific and pharmaceutical interest for their rich phytochemical profiles and traditional utility. Extracts serve as therapeutic agents or adjuvants in folk medicine, used to manage skin conditions (eczema, seborrhea, acne), eye infections [6,7,8], respiratory disorders, gastrointestinal issues, and cardiovascular or urinary tract diseases [8,9,10].

The phytochemistry of the genus is characterized by a wide range of bioactive compounds, including salicylic acid esters, phenolic carboxylic acids, linoleic acid, mucilage, tannins, flavonoids (such as rutin and anthocyanidins), carotenoids, vitamins, and triterpenic saponins [7,10,11]. Several specific compounds serve as chemotaxonomic markers, including violantin, violaquercetoside, the alkaloid violin, and cycloviolacin O2 [12,13,14]. Collectively, these constituents confer significant anti-inflammatory, antioxidant, and antimicrobial properties.

Within the genus, the section *Melanium* forms a distinct taxonomic group of approximately 100–115 annual and perennial taxa, distributed primarily in southern Europe [15,16]. Cytogenetically, this section is defined by extensive polyploidy, aneuploidy, and a high proportion of endemic species [1,5,15,17]. A unique anatomical feature of *Melanium* is the presence of secretory root cells producing methyl salicylate, an ester valued for its analgesic and vasodilatory properties [18].

Several Melanium species are well studied due to their medicinal value. *Viola tricolor* L. and *V.* arvensis Murr. are cosmopolitan taxa found from lowlands to subalpine zones [2,19]. Both are recognized traditionally for treating dermatological, respiratory, digestive, and rheumatic disorders [20,21].

In contrast, *Viola kitaibeliana* Schult. inhabits arid, sandy environments and is considered endangered in regions such as Italy [22]. *V. declinata* Waldst. et Kit. and *V. dacica* Borbás are mountain species, endemic to the Carpathians and protected in Romania [23,24]. *V. declinata* typically grows in high-altitude siliceous grasslands, contributing to ecological stability [25,26]. While *V. declinata* is generally classified as a Carpathian endemic and *V. dacica* as an Alpine–Carpathian–Balkan element, their ranges overlap, leading to occasional taxonomic confusion [25,26,27].

Both *V. declinata* and *V. dacica* show potential as natural sources of anthocyanins [28]. *V. declinata* exhibits a phytochemical profile similar to *V. tricolor*, suggesting comparable pharmacological potential [11,29,30,31]. However, unlike the well-documented *V. tricolor* and *V.* arvensis, data regarding *V. declinata*, *V. dacica*, and *V. kitaibeliana* remain limited. Consequently, this study aimed to provide a comparative assessment of the phytochemical composition and antioxidant potential of these five *Melanium* species.

## 2. Results

### 2.1. Assimilatory Pigments

**Figure 1:**
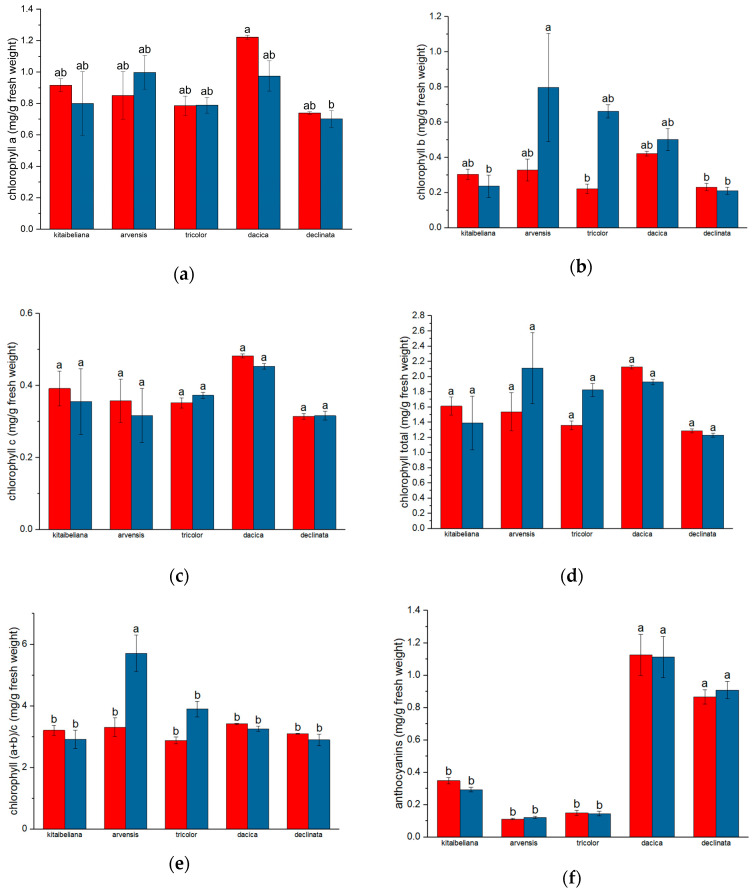
Chlorophyll contents of *Viola* species: red columns—first sampling year, blue columns—second sampling year; (**a**) chlorophyll a, (**b**) chlorophyll b, (**c**) chlorophyll c, (**d**) total chlorophyll, (**e**) chlorophyll a + b to chlorophyll c ratio) and (**f**) total anthocyanins; values are expressed as mg/g fresh weight ± standard error; different letters indicate statistical significance for *p* < 0.05.

The profiling of assimilatory pigments indicated that, across all the studied taxa, carotenoid concentrations generally exceeded those of green chlorophylls (Figure 1). This trend suggests a physiological adaptation to light stress. *Viola dacica*, harvested from alpine meadows, consistently exhibited the highest carotenoid content, recording 0.483 ± 0.006 mg/g in the first year and 0.453 ± 0.008 mg/g in the second year. This was followed by *V. kitaibeliana* (0.392 ± 0.049 mg/g in the first year vs. 0.356 ± 0.091 mg/g in the second year) and *V. arvensis* (0.358 ± 0.06 mg/g in the first year vs. 0.317 ± 0.075 mg/g in the second year). *V. tricolor* and *V. declinata* showed relative stability between years: *V. tricolor* ranged from 0.352 ± 0.014 mg/g (first year) to 0.373 ± 0.008 mg/g (second year), while *V. declinata* remained consistent at 0.315 ± 0.008 mg/g (first year) and 0.316 ± 0.012 mg/g (second year).

The total assimilatory pigment content strongly reflected the solar incidence of the collection sites (alpine meadows, open pastures, and agricultural areas), illustrating the plasticity of the photosynthetic apparatus in response to habitat conditions [32]. The highest total pigment values were observed in species exposed to intense irradiance: *V. dacica* (alpine meadows) peaked at 2.126 ± 0.036 mg/g (first year) and 1.93 ± 0.027 mg/g (second year). Similarly, *V. kitaibeliana* (pastures) reached 1.612 ± 0.105 mg/g (first year), and *V. arvensis* (agricultural area) reached 2.112 ± 0.238 mg/g (second year).

### 2.2. Anthocyanin Compounds

Anthocyanin content varied significantly with floral phenotype (Figure 1f). *V. kitaibeliana*, *V. arvensis*, and *V. tricolor*, which possess multicolored petals (white-cream to dark blue), generally showed lower concentrations. The lowest values were recorded for *V. arvensis* (0.112 ± 0.005 mg/g in the first year vs. 0.116 ± 0.006 mg/g in the second year) and *V. tricolor* (0.15 ± 0.016 mg/g in the first year vs. 0.145 ± 0.014 mg/g in the second year). *V. kitaibeliana* displayed intermediate values, decreasing from 0.349 ± 0.02 mg/g in the first year to 0.293 ± 0.015 mg/g in the second.

Conversely, *V. declinata* and *V. dacica*, which are characterized by intense indigo corollas, contained significantly higher quantities of anthocyanins, marking them as superior sources of these bioactive compounds. *V. dacica* yielded the highest results, with 1.125 ± 0.127 mg/g (first year) and 1.113 ± 0.128 mg/g (second year). *V. declinata* followed, showing a slight year-over-year increase from 0.867 ± 0.044 mg/g (first year) to 0.909 ± 0.054 mg/g (second year).

### 2.3. Phenolic Content

*V. declinata* and *V. dacica*, both adapted to demanding environmental conditions, exhibited the highest phenolic content. For *V. declinata*, hydroalcoholic extraction was most efficient at a 1% plant-to-solvent ratio (95.53 ± 0.33 mg GAE/g). Efficiency decreased at other concentrations: −27% at a 2.5% ratio, −55% at 5%, and −61% at 0.5%. Aqueous extracts followed a similar pattern, peaking at the 1% ratio (~85 mg GAE/g) and declining by 33–63% at other ratios. *V. dacica* showed a comparable trend; hydroalcoholic extracts peaked at 1% (94.81 ± 1.37 mg GAE/g), while aqueous extracts reached 78.61 ± 1.43 mg GAE/g at the same concentration.

**Figure 2:**
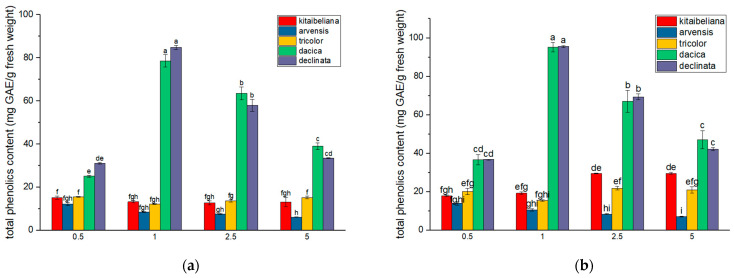
Mean phenolic contents of *Viola* species: (**a**) aqueous extracts; (**b**) hydroalcoholic extracts; values are expressed as mg of GAE/g of dry biomass ± standard error; different letters indicate statistical significance for *p* < 0.05.

The other three species showed lower phenolic levels. *V. arvensis* recorded minimum values, peaking at a 0.5% concentration for both aqueous (11.93 ± 0.40 mg GAE/g) and hydroalcoholic extracts (13.72 ± 0.47 mg GAE/g). *V. kitaibeliana* achieved its highest aqueous yield at a 0.5% ratio (15.02 ± 0.44 mg GAE/g), whereas its hydroalcoholic extracts were most effective at 2.5% and 5% ratios (~29.45 ± 0.17 mg GAE/g) (Figure 2).

### 2.4. Flavonoid Content

**Figure 3:**
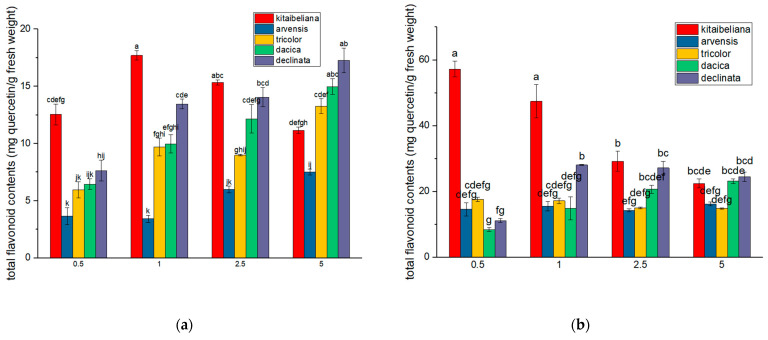
Mean total flavonoid content in aqueous (**a**) and hydroalcoholic (**b**) *Viola* extracts of four concentrations; values are expressed as mg quercetin/g dry biomass ± standard error; different letters indicate statistical significance for *p* < 0.05.

Although *V. declinata* and *V. dacica* dominated in total phenolics, *V. kitaibeliana* was the standout species regarding flavonoid content (Figure 3). For aqueous extracts, the highest flavonoid value was found at a 1% ratio (17.75 ± 0.26 mg QE/g). However, hydroalcoholic extraction proved significantly more effective for this species, peaking at a 0.5% concentration with a substantial yield of 56.61 ± 1.19 mg QE/g. As the plant-to-solvent ratio increased, extraction efficiency steadily decreased: 1% (−16%), 2.5% (−47%), and 5% (−60%).

### 2.5. Scavenging Capacity

The highest antioxidant activity was generally recorded at a 1% plant-to-solvent ratio, coinciding with the peak phenolic yields described in Section 2.3. These samples exhibited approximately 32–33% inhibition rates (approx. 14.80 ± 10.5 mg GAE/g for aqueous and 15.50 ± 0.90 mg GAE/g for hydroalcoholic extracts).

**Figure 4:**
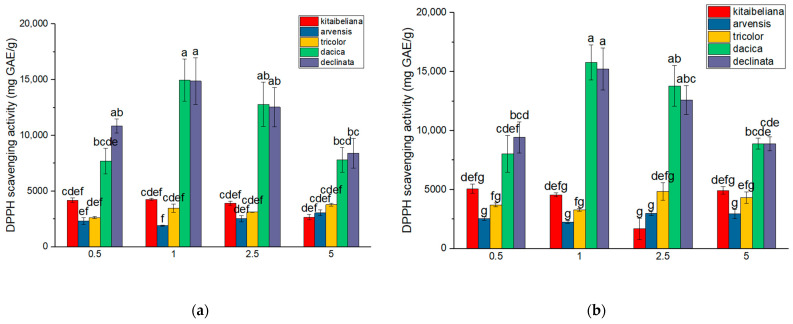
Mean DPPH scavenging capacity mg GAE/g in aqueous (**a**) and hydroalcoholic (**b**) *Viola* extracts of various concentrations; mg of GAE/g dry biomass ± standard error; different letters indicate statistical significance for *p* < 0.05.

**Figure 5:**
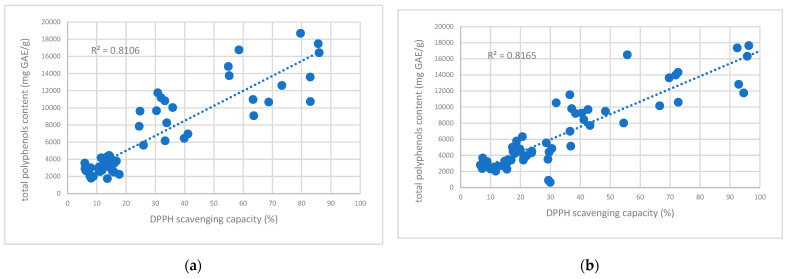
Correlation between DPPH scavenging capacity and total polyphenol content in aqueous (**a**) and hydroalcoholic (**b**) *Viola* extracts.

In the specific case of *V. kitaibeliana*, scavenging capacity was more closely linked to total flavonoid content rather than total phenolics. The highest values for this species were observed at a 0.5% ratio (approx. 5.08 ± 0.19 mg GAE/g; ~26% inhibition). *V. arvensis* displayed the lowest antioxidant potential, with a maximum inhibition rate of only 20% (approx. 3.04 mg GAE/g) obtained at a 5% concentration (Figure 4). A strong, positive correlation (*p* < 0.05) was observed between total phenolic content and antioxidant activity, as measured by 2,2’-diphenyl-1-picrylhydrazyl (DPPH) scavenging capacity (Figure 5).

## 3. Discussion

A quantitative assessment of an assimilatory pigment was carried out using five parameters: chlorophyll a, chlorophyll b, total carotenoids, total assimilatory pigments (chlorophyll a + chlorophyll b + carotenoids), and the chlorophyll-to-carotenoid ratio. All five parameters were evaluated over a 2-year consecutive period.

The amount of chlorophyll pigments in leaves varies with various external habitat and internal plant factors such as the age of the leaves. The type and amount of nutrients vary from one landform to another and from one biocenosis to another, respectively, directly influencing the accumulation of biomass, which is reflected in the increase in the amount of assimilatory pigments [33,34]. Within our results, *V. dacica* individuals consistently recorded higher concentrations of total chlorophyll, chlorophyll a, chlorophyll c, and anthocyanins; however, there were no statistically significant differences compared with other taxa (Figure 1).

The role of carotenoid pigments not only increases the efficiency of photosynthesis but also protects against the negative effects of exposure to high levels of solar radiation [35,36]. The data obtained from total assimilatory pigments correlate with the hypothesis of the adaptive measure of the photosynthetic apparatus. The analysis of anthocyanin compounds was carried out for two consecutive years on plant material collected from the same locations. The reason for this choice was the desire to obtain accurate results regarding the quantity of anthocyanins, considering that the synthesis of these compounds is dependent on the type of soil and its quality. The data obtained points out that the color of the corolla of collected plant material is more white-cream and yellow than blue or indigo. Nutrient type and soil pH have a direct influence on petal color [37]. Moreover, the pH in the cells of the petals determines the structure of the anthocyanins and the light absorption spectrum that influences the perception of color, a pH that is influenced by that of the soil and that correlates with water stress, but also with the natural senescence process of the plant [38,39]. The pattern of colors and their intensity is also due to the expression of genes involved in the metabolic pathway of the synthesis of anthocyanin pigments. The color pattern in the petals occurs because of differential expression of the genes involved, and as a result, clusters of cells appear that give the spot appearance to the petals [37,40]. *Melanium* subgenus is considered to have metallophyte species. For example, *V. tricolor* can grow on soils rich in metal ions (zinc, magnesium), including heavy metals (lead). For this reason, the corolla has a variety of color combinations, and in the presence of iron ions, flowers will have more intense blue-violet colors [41,42,43,44,45].

*V. declinata* and *V. dacica* are present in alpine and subalpine meadows. The intense color may be determined by ecological requirements that make the plant compete for pollinators [45,46,47,48]. Likewise, studies have shown that *V. dacica* can grow on soils rich in metal ions, and can store the metal ions in its roots, like *V. tricolor*, which is another reason that these plants have intensely colored petals [43,44,47]. Considering the fact that *V. dacica* and *V. declinata* grow in similar habitats, we can consider that *V. declinata* can also be a metallophyte species. Regarding the pharmacological potential of anthocyanin pigments, although they are not considered essential nutrients, they are associated with maintaining long-term human health due to their antioxidant effect [46,48]. So far, only a limited screening of biocompounds has been conducted for *V. declinata*, and the presence of anthocyanins and proanthocyanins was highlighted [12].

Phenolics are a complex class of secondary aromatic compounds with a major role in self-protection of the plants against pathogens and insects [49]. Due to these characteristics, plant extracts are used in traditional medicine in different forms and concentrations. Based on this knowledge, we decided to experiment with different types of extracts and concentrations, which are most likely to be used in traditional medicine.

The differences between the values obtained in the analyzed species may be due to the environmental conditions and the type of biocenosis of which they are part. It is known that polyphenols are involved in plant adaptation mechanisms to less favorable abiotic conditions, such as acidophilic or nutrient-poor soils (such as those in alpine areas or polluted soils) [50]. Our data correlate with this hypothesis, meaning that the highest values, for both types of extracts and all four concentrations, were obtained for the plant material collected from alpine and subalpine meadows (Figure 2). *V. declinata* and *V. dacica* are adapted to habitats with demanding requirements: low temperatures and soil with acidic pH and/or poor in nutrients [51,52]. Between these two species, there was no statistically significant difference in the values for polyphenols, which is to be expected, since they grow in similar habitats. 

Compared with the mountain species, the values of total phenolic extraction for *V. tricolor*, *V. arvensis*, and *V. kitaibeliana* did not reveal such drastic differences between plant-to-solvent ratios for both aqueous and hydroalcoholic extracts. Although *V. tricolor*, and sometimes *V. arvensis*, are the most used in pharmacology applications for their biocompounds, our research revealed that the highest value for total phenolic content was obtained from *V. kitaibeliana* extracts compared with these two.

Taking into consideration all our data so far, we can consider that our differences in values for the four extract concentrations are due to the type of solvent, method, and solvent-to-solid ratio that affect the optimum extracting concentration. The quantity and quality of the polyphenols extracted are influenced by temperature, solvent concentration, and pH [53]. Another important step is the method of drying and griding the plant samples. Studies show that extraction from freeze-dried plant material may yield higher levels of phenolic content than in air-dried plant samples [54]. The particle size of the sample is obtained by grinding and solvent-to-solid ratio [55,56,57]. Optimization of the solvent-to-solid ratio is dependent on particle size of the sample and structural components of the cell wall, and we must take into account high costs and solvent wastes, plant material, respectively, and avoidance of saturation effects [54,55,56]. Considering the obtained data for polyphenols, for *V. declinata* and *V. dacica*, where the highest values were observed at a lower plant-to-solvent ratio, it is possible that starting at 2.5% and 5% extract concentration, the saturation effects occur, according to mass transfer principles, given the fact that they contain a high quantity of polyphenolic compounds. Therefore, a lower sample amount and lower particle size can optimize polyphenols extraction [55,56].

Another important factor that we can discuss is the structural components of the cell wall. Phenols are linked to the polysaccharides of the cell walls, can be found in the vacuoles, or are associated with cell nuclei, depending on the composition of both phenols and polysaccharides [57,58]. *V. tricolor* and *V. arvensis* are species that can grow up to 50–200 mm height, due to adaptation to environmental conditions and the flora with which they compete [20,42,59]. Meanwhile the other three species, including *V. kitaibeliana*, collected from a similar Biocoenose with *V. tricolor* and *V. arvensis*, are smaller in size. The structural components needed for wall strength affect the bioavailability of certain compounds, including polyphenols. The phenolic compounds play an important role in wall strength, cell expansion, degradability, and pathogen resistance [57,58]. Due to these interactions between polyphenols and other compounds of the cell wall, the extraction of these biocompounds is directly influenced by the particle size and plant-to-solvent ratio. This possibility may be one of the reasons for the differences between the values and between species of total polyphenols extracted. Polyphenolic compounds are recognized in modern medicine for their anti-inflammatory and antibacterial effects and are used as adjuvants in the treatment of multiple conditions [6]. Given our quantitative results, we can conclude that *V. kitaibeliana* can be an important source of polyphenol extraction. 

Flavonoid compounds are part of the class of polyphenols. They are secondary metabolites involved in plant response to abiotic stress [52,60,61]. Stress factors influence the expression of some genes involved in the biosynthesis of phenols, and of course, flavonoids. Experiments on plants belonging to the species *V. tricolor* revealed the activation of the genes phenylalanine ammonia-lyase (PAL), chalcone synthetase (CHS), flavonoid 3′,5′-hydroxylase (F3′5′H), and flavonoid 3′-hydroxylase (F3′H) in response to silver nanoparticle treatment [62]. Regarding the other species, *V. declinata* and *V. dacica* are also rich in flavonoid compounds, which is consistent with the fact that these are alpine species that grow on nutrient-poor soils and in harsh meteorological conditions. There are studies that discuss the involvement of flavonoid compounds in regulating the relationship of the plant with soil microbiota and the use of some nutrients, such as nitrogen [51,52,63].

For *V. kitaibeliana* and *V. dacica*, the data collected by us are the first data published for total flavonoid content for the chosen extraction methods and plant-to-solvent ratio.

The class of polyphenolic compounds is recognized as having bactericidal, antifungal, anti-inflammatory, and, very importantly, antioxidant properties. The DPPH scavenging capacity is considered particularly important, even more so in the context in which free radicals are considered mediators in cardiovascular and neurological diseases and various types of cancer [63,64,65,66].

Taking into account the research data obtained for the total polyphenols, we can observe that the highest DPPH scavenging capacity for both aqueous and hydroalcoholic *Viola* extracts was registered for *V. declinata* and *V. dacica* compared with the other species (Figure 2 and Figure 4).

The differences in the values of the results obtained both between species and between populations of the same species can also occur due to the quality of the plant material at the time of harvesting, as well as the stress factors acting on the plant. For example, experiments on *V. tricolor* revealed an increase in the values recorded for the activity of antioxidant enzymes such as superoxide dismutase, catalase, L-ascorbate peroxidase, and guaiacol peroxidase, in response to different polluting factors [41,61,62].

Since *V. declinata* and *V. dacica* occupy habitats with difficult environmental conditions, such as alpine meadows and scree [67,68], it is expected that they biosynthesize compounds that offer them competitive adaptations, and, subsequently, increased antioxidant activity.

The data collected in our research are the first data published for *V. kitaibeliana*, *V. declinate*, and *V. dacica* regarding the DPPH scavenging capacity for the chosen extraction methods and plant-to-solvent ratio.

## 4. Materials and Methods

**Figure 6:**
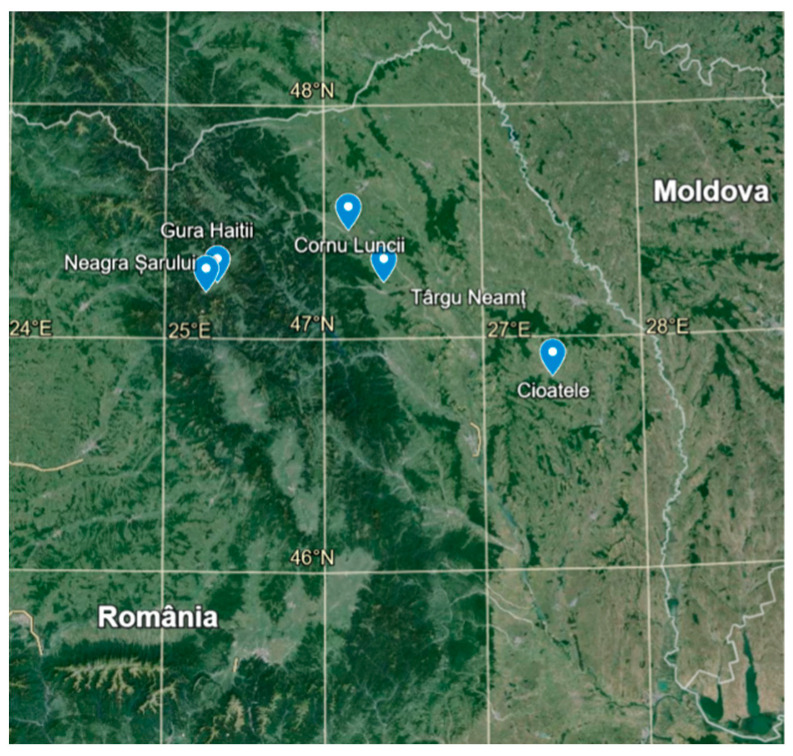
Plant material harvesting locations.

The plant material was collected from spontaneous flora (Figure 6, Table 1) during the flowering phenophase from the North-East of the Romanian: Carpathians meadows (*V. declinata* and *V. dacica*) and from agricultural areas (*V. tricolor*, *V. arvensis*, *V. kitaibeliana*). A randomized grid method was used to select sampling sites by overlaying a grid (10 m^2^ divided into 1 m^2^ squares), then plants were collected from randomly assigned grid intersections (approximately 10 individuals/intersection).

The selected plants had apparently healthy vegetative organs, with most of the leaves and flowers open, free of senescence, and intact (no tears or marks left by herbivorous animals). 

The assimilatory pigments were extracted from fresh leaves, using 85% acetone (Carl Roth GmbH + Co. KG, Karlsruhe, Germany), and analyzed at wavelengths of 663 nm, 645 nm, and 472 nm [69]. 

The total anthocyanin pigments were extracted from fresh flowers in 95% ethyl alcohol (Carl Roth GmbH + Co. KG) with 0.1 N HCl added to create an acid environment to preserve the pigments and quantified spectrophotometrically at 515 nm [70,71].

Extracts were prepared by mixing a 1:9 ground freshly frozen plant material to solvent ratio in 15 mL polyethylene tubes. Total phenols and total flavonoids were analyzed from aqueous and 50% ethanolic extracts (0.5%, 1%, 2.5% and 5% *w*/*v*) extracted at room temperature (20–22 °C), in the dark for 24 h. Air-dried plant material was used, containing the aerial part of the plants without flowers or roots, which was mechanically ground. Conventional methods of analysis [72,73,74,75] were used for the quantitative analysis of total phenolics and flavonoids. Briefly, the total phenolic content was assessed as follows: 0.1 mL of extract was mixed with Folin–Ciocalteu reagent (Merck, Kenilworth, IL, USA) and incubated for 5 min, then Na_2_CO_3_ 7.5% was added, and 90 min incubation followed. The results were calculated referring to 760 nm absorbance and, according to a calibration curve, were expressed as gallic acid equivalents per gram of dry weight. The total flavonoid content was evaluated by the absorbance at 510 nm of extracts reacting with 5% NaNO_2_ and 10% AlCl_3_ (Carl Roth GmbH + Co. KG). The results were expressed according to a calibration curve as quercetin equivalents per gram of dry weight.

Estimation of the antioxidant activity was carried out using the 2-diphenyl-1-picrylhydrazyl method [76,77].

Statistical data processing was performed using OriginPro 9.4. Statistical methods applied were the assessment of value distribution, then analysis of variance of means using ANOVA tests, accompanied by Tukey post hoc testing for *p* < 0.05; correlation was assessed using the Spearman correlation coefficient.

## 5. Conclusions

The comparative assessment of five *Viola* L. species from the subgenus Melanium—*V. dacica*, *V. declinata*, *V. tricolor*, *V. arvensis*, and *V. kitaibeliana*—demonstrates clear interspecific differences in secondary metabolites with pharmaceutical relevance. The Carpathian endemics *V. dacica* and *V. declinata* consistently exhibited the highest levels of total phenolics and anthocyanins across extraction conditions, which strongly and positively correlated with DPPH radical scavenging capacity, underscoring their antioxidant potential. In contrast, the widely used medicinal taxa *V. tricolor* and *V. arvensis* yielded comparatively lower amounts of these bioactive compounds under identical protocols. Notably, *V. kitaibeliana*—sourced from lowland habitats—showed the greatest flavonoid contents (particularly in 50% ethanolic extracts at lower plant-to-solvent ratios), highlighting this lesser-studied species as a promising source of flavonoid-rich extracts. Extraction efficiency varied with solvent and plant-to-solvent ratio; for phenolics in *V. dacica* and *V. declinata*, 1% (*w*/*v*) hydroalcoholic extracts maximized yields, whereas saturation effects likely reduced recovery at higher loadings.

Collectively, these results provide the first quantitative, side-by-side phytochemical baseline for *V. dacica*, *V. declinate*, and *V. kitaibeliana*, expand the phytochemical evidence base beyond the traditionally referenced *V. tricolor* and *V. arvensis*, and point to underutilized taxa with strong antioxidant profiles of potential value to phytopharmaceutical development. Future work should (i) resolve compound-level profiles (e.g., targeted LC–MS/MS of dominant phenolic and anthocyanin constituents), (ii) optimize extraction parameters using formal design-of-experiments to balance yield and solvent economy, and (iii) validate bioactivity in relevant cellular and in vivo models, while considering conservation and sustainable sourcing of the endemic Carpathian species.

## Figures and Tables

**Table 1:** Types of biocenoses and landforms from which the plant material was collected (pp—population).

Species	Plant Population	Biocoenose	Landform
*V. kitaibeliana*	pp. 1	hayfield, agricultural area	plateau ≈365 m above sea level
	pp. 2	agricultural area	plateau ≈450 m above sea level
	pp. 3	pasture	low hilly area ≈122 m above sea level
*V. arvensis*	pp. 1	agricultural area	plateau ≈365 m above sea level
	pp. 2	pasture, agricultural area	low hilly area ≈122 m above sea level
	pp. 3	agricultural area	plateau ≈450 m above sea level
*V. tricolor*	pp. 1	agricultural area	plateau ≈365 m above sea level
	pp. 2	agricultural area	plateau ≈450 m above sea level
	pp. 3	agricultural area	plateau ≈550 m above sea level
*V. dacica*	pp. 1	alpine meadow	Călimani mountains ≈1.043 m above sea level
	pp. 2	alpine meadow	Călimani mountains ≈1.150 m above sea level
	pp. 3	alpine meadow	Călimani mountains ≈1.100 m above sea level
*V. declinata*	pp. 1	alpine meadow	Călimani mountains ≈1.043 m above sea level
	pp. 2	subalpine meadow	Călimani mountains ≈962 m above sea level
	pp. 3	alpine meadow	Călimani mountains ≈1.100 m above sea level

## Data Availability

The original contributions presented in this study are included in the article. Further inquiries can be directed to the corresponding author.

## Abbreviations

The following abbreviations are used in this manuscript:

DPPH	2,2-diphenyl-1-picrylhydrazyl
GAE	Gallic acid equivalents
pp	Population
